# Empowering Public Health Pharmacy Practice—Moving from Collaborative Practice Agreements to Provider Status in the U.S.

**DOI:** 10.3390/pharmacy9010057

**Published:** 2021-03-09

**Authors:** Alina Cernasev, Meghana Aruru, Suzanne Clark, Komal Patel, Natalie DiPietro Mager, Vaiyapuri Subramaniam, Hoai-An Truong

**Affiliations:** 1 Department of Clinical Pharmacy and Translational Science, University of Tennessee Health Science Center College of Pharmacy, 301 S. Perimeter Park Dr., Suite 220, Nashville, TN 37211, USA; acernase@uthsc.edu (A.C.); kpatel64@uthsc.edu (K.P.); 2 Health Analytics Network, LLC, Pittsburgh, PA 15237, USA; 3 Center for Teaching and Learning, California Northstate University College of Pharmacy, 9700 West Taron Drive, Elk Grove, CA 95757, USA; suzanne.clark@cnsu.edu; 4 Department of Pharmacy Practice, Ohio Northern University College of Pharmacy, 525 S Main St, Ada, OH 45810, USA; n-dipietro@onu.edu; 5 Washington Metropolitan Society of Health-System Pharmacists, 11160 Veirs Mill Road Suite LLH-18, Box 332, Silver Spring, MD 20902, USA; vpuris@gmail.com; 6 School of Pharmacy & Health Professions, University of Maryland Eastern Shore, 1 College Backbone Road, Princess Anne, MD 21853, USA; htruong@umes.edu

**Keywords:** pharmacists, collaborative pharmacy practice agreement, provider status for pharmacists

## Abstract

This article describes the history and evolution of pharmacist-physician collaborative practice agreements (CPAs) in the United States with future directions to support pharmacists’ provider status as the profession continues to evolve from product-oriented to patient-centered care and population health. The pharmacy profession has a long history of dispensing and compounding, with the addition of clinical roles in the late 20th century. These clinical roles have continued to expand into diverse arenas such as communicable and non-communicable diseases, antimicrobial stewardship, emergency preparedness and response, public health education and health promotion, and critical and emergency care. Pharmacists continue to serve as integral members of interprofessional and interdisciplinary healthcare teams. In this context, CPAs allow pharmacists to expand their roles in patient care and may be considered as a step towards securing provider status. Moving beyond CPAs to a provider status would enable pharmacists to be reimbursed for cognitive services and promote integrated public health delivery models.

## 1. History and Development of Collaborative Practice Agreements (CPAs)

The profession of pharmacy has a long history of dispensing and compounding with an increasing emphasis on clinical services in the late 20th century. Pharmacy as a clinical profession expanded its role in public health through regulatory, licensure, and cultural changes, including the development of collaborative practice agreements (CPAs) in the late 20th and early 21st centuries.

In the past, pharmacists in the United States (US) often provided a range of compounding, medicinal advice, and other services, based on their knowledge of pharmacognosy and use of natural products to compound medicinal products. Also, during the 19th and early 20th centuries, some physicians were selling medicines directly to patients, which put physicians in competition with apothecaries, druggists, chemists, and pharmacists. With the development of the pharmaceutical industry and the production of drugs, a narrower vision of the profession arose after the Second World War [1]. This led to a post-war phase of constriction of professional roles more limited to “count and pour” services. The Durham-Humphrey Amendment of 1951 and the Federal Food, Drug, and Cosmetic (FDC) Act of 1983 led to the legal separation of prescribing (physicians) and dispensing (pharmacists). Prescription drugs were differentiated from non-prescription or over-the-counter drugs; it also became illegal for pharmacists to refill medications without authorization by a physician [2,3].

During this same time, the American Pharmaceutical Association (now the American Pharmacists Association or APhA) developed a Code of Ethics in 1952 that stated pharmacists were not allowed to discuss the therapeutic effects or composition of a prescription with a patient—that role was reserved for the prescriber (physician or dentist) [1,2]. This resulted in a period of time during which a customer could leave a pharmacy with a prescription bottle, but the label may not have included the name of the drug, nor would the pharmacist be allowed to discuss with the patient the contents or intended use of the prescription. Instead, the patient would be referred back to the physician or other prescriber for answers to their questions. Similarly, during this time, pharmacists were employed in hospitals, but their duties were narrowly focused on products—dispensing, compounding, and formulary development.

Soon after this period, pharmacists’ roles were expanding in selected areas. In the 1960s, pharmacists in the Indian Health Service (IHS) began assuming an active role in drug therapy management. The IHS is the part of the United States Public Health Service (USPHS) that focuses on service in Native American and tribal populations throughout the contiguous US and its owned territories [4]. The first Collaborative Practice Agreement (CPA) was developed by the IHS under a grant from the National Center for Health Services Research in 1973. Specially trained pharmacists provided drug therapy management services in collaboration with physicians. A 1-year evaluation found that physicians judged the quality of care of this program to be satisfactory and patient acceptance was excellent [5]. Since the 1970s, many CPAs have been initiated in the US and a recent review evaluated the characteristics of CPAs across the US [6]. As of 2016, there were 50 states with active CPAs. CPAs vary by state with respect to pharmacist-initiated prescribing activities; there is little uniformity between states regarding pharmacists’ ability to commence, modify, monitor, and discontinue medication therapies, along with heterogeneity in their ability to order laboratory tests [6].

Also in the mid-1950s, US pharmacists embraced patient care roles that provided opportunities in subsequent decades for the profession to shift from dispensing to a more clinically driven practice [7]. The term ‘clinical pharmacy’ reflects the notion that the duties of a ‘regular’ pharmacist were restricted to “count and pour”. In other words, the pharmacist was viewed as a profession that existed outside the healthcare system and as a provider of medications prescribed and communicated by physicians. However, this limited view of the profession was never the norm, and pharmacists have provided clinical services such as patient education, communication, and therapeutic guidance throughout the history of the profession. Higby, (2002) traced the evolution of the modern concept of clinical pharmacy to the 1960s, during which both pharmacists and various institutions were instrumental in moving the profession towards clinical practice [1].

Subsequent regulations, including the federal Omnibus Budget Reconciliation Act of 1990 (OBRA-90), addressed pharmacy services in federally funded and state-managed Medicaid programs [8]. In the US, Medicaid is a joint federal and state program for low-income individuals and families. Most Medicaid enrollees lack access to other affordable forms of health insurance. The program is governed by Title XIX of the Social Security Act and a large body of federal regulations, which define federal Medicaid requirements, state options, and authorities. The Centers for Medicare and Medicaid Services (CMS) within the Department of Health and Human Services (HHS) is responsible for implementing laws governing Medicaid. The CMS recognized that proper drug use was in the best interest of Medicaid recipients, and that pharmacists could play a role in improving drug use through review of medications and prescription counseling. Thus, the OBRA-90, which took effect in 1993, required pharmacists to conduct prospective drug use review (PDUR) of Medicaid prescriptions, and to ‘offer’ to discuss medications with patients at the point-of-sale. This Act helped codify pharmacy services in integral federal health programs.

Pharmacists’ foray into clinical roles led to an urgent need to enhance the training of future pharmacists. As a result, the Doctor of Pharmacy (Pharm.D.) degree was created and designed to parallel to the Doctor of Medicine and other clinical Doctoral degrees [9]. The first Pharm.D. programs were initiated in the 1950s; more programs were developed over recent decades [10]. In the US, Pharm.D. programs are accredited by the Accreditation Council for Pharmacy Education (ACPE).

By the 2000s, all US pharmacy schools and colleges had adopted the Pharm.D. as the universal standard for pharmacy education. Accordingly, the Bachelor of Pharmacy (PharmBS) degree as the professional degree for pharmacy in the US was phased out. In addition, accelerated Pharm.D. programs were developed to allow students to graduate with three years of professional training, following completion of undergraduate prerequisite courses. PharmBS graduates prior to the advent of Pharm.D. degrees were grandfathered in as licensed (registered) pharmacists and use Registered Pharmacist or RPh to denote their status. Pharm.D. and PharmBS pharmacists must comply fully with US Federal and State pharmacy laws. For example, continuing education training (CE hours) is a requirement for maintaining licensure for both PharmBS and Pharm.D. pharmacists [11]. Expansion of clinical roles brought further regulatory and practice opportunities, as exemplified by the development of Pharmaceutical Care and the Medicare Modernization Act of 2005.

In the early 2000s, the US Senate and House of Representatives passed legislation that recognized pharmacists as healthcare providers for Medicare patients at the national level [12]. This has helped advance the pharmacy profession towards greater integration into healthcare teams. Healthcare costs continue to rise in the US and in 2019, healthcare spending grew by 4.6%, reaching $3.8 trillion or $11,582 per person [13]. There is an urgent need to mitigate costly care and simultaneously improve the quality of care.

Pharmacists are well-positioned to provide cost-effective care and effect medication adherence that has a direct impact on patient outcomes. As an example, the Asheville Project, a quasi-experimental, longitudinal pre-post cohort study launched in 1997 in 12 community pharmacies in Asheville, North Carolina led to improved adherence, reduced adverse events, and ultimately decreased overall healthcare costs) [14,15].

## 2. Pharmaceutical Care and Medication Therapy Management (MTM)

During the 1990s, the emergence of “pharmaceutical care” provided the terminology to capture complex concepts of clinical care provision by pharmacists and is highly relevant even today. Pharmaceutical care was defined by Hepler and Strand as “the responsible provision of drug therapy for the purpose of achieving definite outcomes that improve a patient’s quality of life” [16]. The core tenet of pharmaceutical care is to maximize a patient’s therapeutic outcomes by utilizing pharmacists’ skills and knowledge [17,18]. In the past three decades, this concept has gained acceptance and has been implemented in many countries [19,20].

Pharmaceutical care has been presented in the last two decades as a model for pharmacists to develop a therapeutic plan that yields enhanced patient outcomes [17]. To ensure that pharmaceutical care goals are achieved, and mitigate medication-related issues, pharmacists collaborate closely with the healthcare team. To date, the pharmaceutical care model used in different US healthcare systems is composed of a range of pharmacists’ services that contribute directly to patient outcomes [21]. Further, the scope of pharmacy practice is increasingly integrated into team-based care and is responsible for medication management [12].

With the advent of the Medicare Modernization Act (MMA) of 2003, prescription medications for the Medicare program (care for the elderly and disabled) which were previously not covered would now be reimbursed under Medicare Part D. (Parts A, B and C covered hospital, inpatient, and managed care services). This change was largely brought about due to the changing US demographics, with an increase in the senior population and the need to ensure that this population had coverage through the federal Medicare program to access needed prescription medications.

In recognition of the fact that medication monitoring in this population was crucial to avoid therapeutic duplications, minimize adverse events, and improve medication adherence, the term Medication Therapy Management (MTM) was coined. MTM is defined as “…*distinct service or group of services that optimize therapeutic outcomes for individual patients*.” MTM can be provided by pharmacists, or another qualified healthcare provider’s scope of practice, independently or together with a medication product [22]. In addition to the MTM consensus definition by 11 professional pharmacy organizations in 2005, the APhA and the National Association of Chain Drug Stores (NACDS) collaborated to develop a MTM Core Elements Service Model that include five core elements:

(1) medication therapy review (MTR),
(2) personal medication records (PMR),
(3) medication-related action plan (MAP),
(4) intervention and/or referral, and
(5) documentation and follow-up [23]

Although all five core elements are essential to providing MTM services, their order and delivery may be adapted to best serve patient needs. Pharmacists can provide MTM in all practice settings to optimize medication use towards improved clinical outcomes.

A core element of MTM is medication therapy review (MTR), which is a “systematic process of collecting patient-specific information, assessing medication therapies to identify an actual or potential medication-related problem(s).” Problems are then prioritized to create a plan of action. MTR could take the form of a comprehensive medication management or targeted medication review. During MTM consultation, including comprehensive or targeted medication review, pharmacists make recommendations for changes in therapy to a prescriber for implementation. However, pharmacists may change and/or initiate drug therapy under specific protocols if they work with prescribers under a Collaborative Practice Agreement (CPA) or Collaborative Drug Therapy Management (CDTM) [24,25].

The following sections present CPAs as a means of empowering the profession and a step toward provider status recognition to achieve the profession’s potential in the provision of patient-centered care and improving public health.

## 3. Empowering the Pharmacy Profession: CPAs

**Figure 1:**
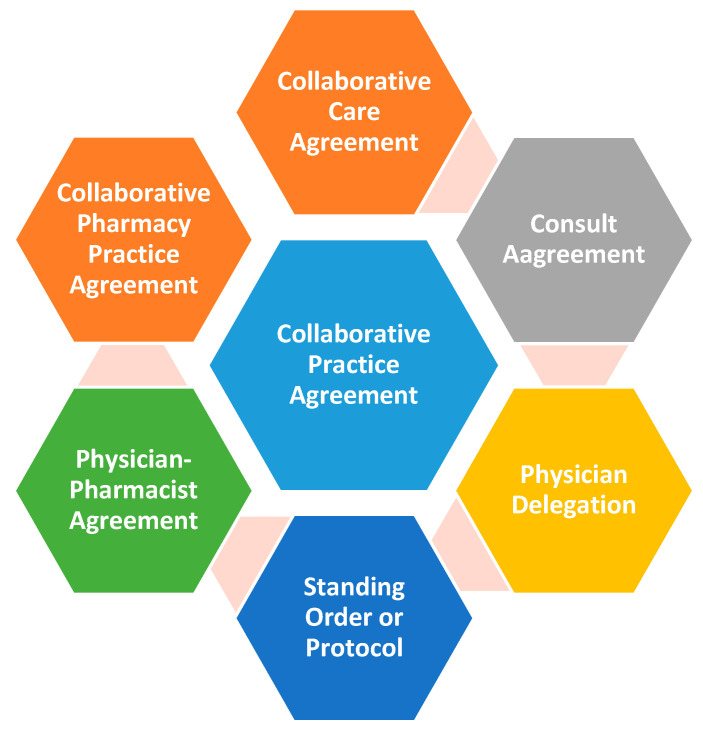
Collaborative Practice Agreement (CPA) terms in use.

CPAs are defined as “an agreement between one or more physicians and pharmacists wherein qualified pharmacists working within the context of a defined protocol are permitted to assume professional responsibility for a variety of functions, including patient assessment; ordering drug therapy—related laboratory tests; and selecting, initiating, monitoring, continuing and adjusting drug regimens” [12]. CPAs include disease-specific education or medication management to achieve desirable clinical outcomes [26]. The term CPA varies by state and several terms are used to describe these arrangements (Figure 1) [25].

Leveraging and maximizing CPAs may lead to enhanced relationships with other healthcare providers, allowing pharmacists to contribute significantly to safety and efficiency of medication use towards improved adherence, better outcomes, and reduced healthcare costs [27]. CPAs can be used to delegate a wide variety of patient care functions including, but not limited to, modifying prescriptions, ordering laboratory tests, chronic care management, refill authorizations, and formulary management. CPAs are negotiated between the collaborating physician and pharmacist. These may vary by state depending upon the state’s practice laws, practice environment, and the practitioners’ preferences. Hammond R, et al. (2003) describe the differences in CPAs across various states (See Table S1) [12].

CPAs signify trust in the professional judgement between the collaborating physician and pharmacist, ensure continuity-of-care for patients, enhanced patient-physician-pharmacist relationships, and allow for efficient workflows. More importantly, CPAs allow each type of practitioner to utilize their own skills and leverage the skills of the other practitioners to provide direct patient care.

Consider the following example (Box 1):

Box 1 **CPA utility in improving continuity-of-care.**

Before CPAs existed, a physician could refer a patient to a pharmacist for measuring their vitals, adjusting their medications, counseling, and to discuss adherence. The pharmacist would then call the physician with their recommendations and the physician would be able to make those changes through a prescription change. However, this would likely result in several phone calls and discussions between the physician and pharmacist, ultimately leading to less desirable patient care through either delays or unavailability of selected prescriptions.

Once CPAs came into existence, the pharmacist (depending upon state law and extent of the CPA) would be able to make those medication regimen changes and fill the prescription for the patient while they wait at the pharmacy. The change would be noted in the electronic health record that would be shared with the physician to refer to at the patient’s future visit. This not only leads to efficiency in the workflow by reducing the number of calls between the practitioners, but also provides direct patient care and ensures that care optimization is not delayed or deferred for a later time.

Examples of innovative applications of CPAs have extended to telehealth where community pharmacists play key roles in hypertension management [28]. Likewise, Awdishu et al. discussed the importance and the positive effect of the CPAs on the pharmacy practice in academic health systems [29]. A qualitative study exploring medical doctors’ perspective towards pharmacists’ delivery of clinical services in Utah revealed that the healthcare system needs to develop protocols to nurture a more robust collaborative practice between medical doctors and pharmacists. The participants also acknowledged the role of pharmacists as a vital resource in developing interventions [30]. For instance, Lagisetti et al. described the advantages of having a pharmacist on the healthcare team for changing the therapeutic regimen to buprenorphine for pain management [28].

CPA benefits culminated in the 2000s, when pharmacists were authorized to administer vaccines. Since then, per a recent report from APhA, pharmacists in all 50 states, as well as the District of Columbia, and Puerto Rico can administer immunizations [31,32]. Pharmacists have embraced their roles as immunizers in their communities and contributed to expanded immunization rates [33,34]. A recent report from the Centers for Disease Control and Prevention (CDC) indicated that 28% of the US adults received immunization at a pharmacy [35]. The CPA process is directly responsible for this change, because community pharmacists cannot administer immunizations without a CPA agreement authorizing this capability.

CPAs are not a requirement for collaborative care; however, they serve to improve the efficiency and effectiveness of collaborative care delivery through increased access, improved collaborations and cooperation, and recognize pharmacists’ knowledge as medication experts. Despite the benefits of CPAs, some states have restrictive language that does not fully leverage the benefits of CPAs for rural and other communities.

Barriers to fully implement CPAs in community settings may include restrictive language such as limits on the number, types, and qualifications of pharmacists who may enter a CPA. The National Alliance of State Pharmacy Associations (NASPA) recommends the use of permissive language to ensure that CPA are more accessible to physicians and pharmacists towards improving patient care provision [36]. The CDC, NASPA, and Change Lab Solutions developed a toolkit for pharmacists as a resource to help in developing CPAs [35]; practitioners may find the toolkit useful in developing new CPAs, or enhancing existing ones [36]. While CPAs are demonstrated to improve collaboration and recognize pharmacist expertise, they are impeded by lack of cognitive reimbursements for pharmacists [29]. Hence, the next step would be to consider moving beyond CPAs to conferring provider status to pharmacists that would enable them to freely collaborate and work with physicians and other healthcare providers and be reimbursed for their services.

## 4. Development of Public Health Pharmacy: Towards Provider Status

CPAs have empowered the pharmacy profession and served as an important step enabling pharmacists to build their skills in public health education, wellness, and immunizations, while allowing educational institutions and professional pharmacy organizations to conduct education, training, and capacity development. To fully facilitate the profession’s potential in the provision of patient-centered care and improve public health, the next steps would be to phase out CPAs and recognize pharmacists as healthcare providers (with explicated roles, responsibilities, and reimbursements) to promote the synergistic collaboration and cooperation that would serve to provide enhanced public health services across the country.

Provider status enables healthcare providers to bill payors (e.g., insurance companies) for reimbursement [37]. Pharmacists’ professional training and competence is often greater than their legal scope of practice [38]. Federal and state laws could authorize pharmacist provider status, yet this increase in scope of practice has yet to be realized broadly. Similarly, reimbursement for such services is not consistently applied across public and private payors, hindering pharmacists’ ability to expand the scope of services provided [38]. Pharmacists’ involvement in healthcare teams has been shown to decrease total health expenditures, decrease unnecessary care, and decrease societal costs [39]. By enabling provider status for pharmacists, healthcare systems can actualize these benefits.

## 5. Actions during COVID-19

During the COVID-19 pandemic, 13 pharmacy organizations advocated for pharmacists to be granted provider status. While this petition was not approved, pharmacists have continued to serve their communities through COVID-19 testing, ensuring continuous supply of essential medications, and preparing for the COVID-19 vaccine rollout (For a state-by-state CPA and provider status, please see Table S1).

At times, public health emergencies have resulted in states expanding the scope of practice for pharmacists, either through emergency legislation or state governors’ executive orders [38,39]. Pharmacists’ roles to provide patient care during COVID-19 were recognized by the US Department of Health and Human Services (HHS) guidance issued in May 2020 of the Public Readiness and Emergency Preparedness (PREP) Act [40]. The PREP Act authorizes pharmacists to order and administer point-of-care testing for SARS-CoV-2 for the duration of the public health emergency [40]. This affords states’ opportunities to expand regulations allowing point-of-care testing by licensed pharmacists and identify appropriate reimbursement procedures.

## 6. Future Directions

The number and type of CPAs have expanded over the years. Pharmacists continue to provide expanded, clinical services through CPAs in a variety of settings and the evidence on clinical services provided by pharmacists continues to grow. The evolving nature of healthcare and increasing numbers of aging populations in the US and globally necessitate a continued evolution of the pharmacy profession to encompass public health, emergency preparedness, and population health activities that would empower them to deliver patient-centered care.

Healthcare costs in the US have reached $3.8 trillion in 2019 and are projected to grow at an annual rate of 5.4% during 2019–2028 and reach $6.2 trillion by 2028 [41]. Healthcare providers are increasingly looking to pharmacists to monitor drug therapy, manage adherence towards cost-effectiveness, and improve patient outcomes. In the COVID-19 pandemic and other emergencies, pharmacists have become instrumental in conducting tests, providing education, ensuring medication supplies, and managing medications therapy.

In 2000, the Institutes of Medicine published the seminal work on patient safety “To Err is Human” that examines medical and pharmaceutical errors in healthcare. The 2001 “Crossing the Quality Chasm: A New Health System for the 21st century” made an urgent call for fundamental change to close the gap in quality of healthcare services provided. In the 21st century, the percentage of patients who take several drugs for chronic diseases continue to increase. Based on existing trends, there would be an increase in the number of patients lacking adequate access to care, receiving suboptimal, inappropriate, or unnecessary drug therapy as costs continue to rise. Pharmacists are capable, willing, and prepared to address these challenges and impact patient outcomes. Through CPAs and other interdisciplinary settings, pharmacists have amply demonstrated their value in improving effectiveness, safety, and efficiency of medication therapy. It is therefore time to incorporate this key element of healthcare by recognizing these contributions and empowering pharmacists through provider status. This will place pharmacists as integral members of healthcare teams as healthcare provision continues to evolve in the 21st century and beyond.

## Supplementary Materials

The following are available online at https://www.mdpi.com/2226-4787/9/1/57/s1, Table S1: Pharmacy CPA and provider status by state.
