# Supplementary material for: Empowering Public Health Pharmacy Practice—Moving from Collaborative Practice Agreements to Provider Status in the U.S
Source: Pharmacy (Basel). 2021 Mar 9;9(1):57. doi: 10.3390/pharmacy9010057 (PMC8005938; doi:10.3390/pharmacy9010057)
Supplement: Supplementary file 1 [file pharmacy-09-00057-s001.pdf]

Table S1: Pharmacy CPA and Provider status by state

State	Qualified Pharmacist permitted to Initiate, Modify, and/or Discontinue Therapy	Additional Requirements	Implementation (Updated 01/05/2021)	COVID-19 Action (Updated on 01/05/2021)	Pharmacist Provider Status (Updated 1/5/2021)
AL	Yes	No	Yes	Yes. Emergency order was declared on March 13, 2020. Allowed healthcare facilities to implement “alternative standards of care.” Vaccine authorized to administration (No age restriction).	Yes
AK	Yes	No	Yes	Yes. Emergency order was declared on March 11, 2020. Authorized out-of-state pharmacists to practice in Alaska with an emergency permit under certain conditions, no need to take the MPJE. Vaccine authorized to administration.	No
AZ	Yes	No	Yes. Any licensed pharmacist, in any practice setting, can participate in a CPA. No extra training required.	Yes. Emergency order was declared on March 11, 2020. Authorized pharmacists to administer any vaccine to persons of any age without a prescription order during a public health emergency.	Yes
AR	Yes	Yes	Yes, with additional requirements.	Yes. Emergency order was declared on March 11, 2020. Vaccine authorized to administer. No age restriction.	Yes
CA	Yes	Yes	Yes. Specific certification program training requires CPA	Yes. Emergency order was declared on March 04, 2020. DCA ordered waiving restrictions on pharmacists ordering and collecting specimens for COVID-19 tests. Vaccine authorized to administration (Age restriction: 3+).	Yes
CO	Yes	Yes	Yes	Yes. Emergency order was declared on March 11, 2020. Allow pharmacists to conduct COVID testing and administer vaccines.	Yes
CT	Yes	No	Yes	Yes. Emergency order was declared on March 10, 2020. Vaccines authorized to administer (Age restriction: 18+).	Yes
DE	No	No	No	Yes. Emergency order was declared on March 12, 2020. Directs health care providers who are providing testing to do free of charge, regardless of the person’s insurance coverage. Vaccines authorized to administer.	No
DC	Yes	Yes	Yes, with additional requirements.	Yes. Mayor's Emergency order was declared on March 11, 2020. Vaccines authorized to administer.	No
FL	Yes	Yes	Yes, with specific state requirements	Yes. Emergency order was declared on March 09, 2020. Vaccines authorized to administer (Adults only).	Yes
GA	Yes	Yes	Yes. Board of Pharmacy involvement in agreements.	Yes. Emergency orders was declared on March 14, 2020. Emergency rule authorized delivering prescriptions by mail upon patient request, under certain conditions. Emergency rule authorized remote order verification under certain conditions. Authorized to administer vaccines.	No
HI	Yes	Yes	Yes. Required appropriate training approved by credentialing body.	Yes. Emergency order was declared on March 05, 2020. Authorized out-of-state licensed physicians, physician assistants, and nurses to practice in Hawaii– pharmacists not included. Authorized to administer vaccines. (Age restriction: 18+).	No

State	Qualified Pharmacist permitted to Initiate, Modify, and/or Discontinue Therapy	Additional Requirements	Implementation (Updated 01/05/2021)	COVID-19 Action (Updated on 01/05/2021)	Pharmacist Provider Status (Updated 1/5/2021)
ID	Yes	No	Yes	Yes. Emergency order was declared on March 13, 2020. Implemented process for rapid licensure/registration of new support staff. Authorized to administer vaccines. (Age restriction: 6+).	Yes
IL	Yes	No	Yes. No state mandated requirements for extra training for pharmacist.	Yes. Emergency orders was declared on March 09, 2020. Authorized to administer vaccines. (Age restriction: 14+ with prescription/protocol).	No
IN	Yes	No	Yes	Yes. Prohibited price gouging. Allowed out-of-state licensed health care providers to practice in Indiana. Pharmacist: Technician ratio increased from 6:1 to 8:1. Authorized to administer vaccines. (Age restriction: 11+).	Yes
IA	Yes	No	Yes	Yes. Allowed certain healthcare providers whose license is inactive or lapsed to practice in Iowa. Did NOT include pharmacists. Issued Proclamation of Disaster relief. Authorized to administer vaccines. (Age restriction: 6+ for emergency vaccines).	Yes
KS	Yes	No	Yes	Yes. Emergency order was declared on March 12, 2020. Recommended pharmacies encourage customers use drive-throughs, when available, separate customers in line, put more space between customers and pharmacy staff at counter. Authorized to administer vaccines. (Age restriction: 12+).	Yes
KY	Yes	No	Yes	Yes. Emergency order was declared on March 17, 2020. Allowed pharmacists to practice remotely utilizing a closed network pharmacy software as long as there was a pharmacist present to conduct final verification and in-patient counseling Authorized to administer vaccines.	Yes
LA	Yes	No	Yes	Yes. Authorized an extension of the expiration date for certain education requirements. Authorized to administer vaccines. (Age restriction: 17+).	Yes
ME	Yes	Yes	Yes. Board of Pharmacy approves Pharmacist listed in agreement. Pharmacist must have state required training for CPA.	Yes. Emergency order was declared on March 15, 2020. Implemented emergency policies as informed by CDC guidance for employers and information for pharmacies from APhA and ASHP. Authorized to administer vaccines. (Age restriction: 18+).	No
**State**	**Qualified Pharmacist permitted to Initiate, Modify, and/or Discontinue Therapy**	**Additional Requirements**	**Implementation (Updated 01/05/2021)**	**COVID-19 Action (Updated on 01/05/2021)**	**Pharmacist Provider Status (Updated 1/5/2021)**

MD	Yes	Yes	Yes. Board may determine if each pharmacist has the basic qualifications to perform under this Physician-Pharmacist Agreement. The Board of Pharmacy must approve each Pharmacist listed in the agreement.	Yes. Emergency order was declared on March 05, 2020. Implemented emergency policies as informed by CDC guidance for employers and information for pharmacies from APhA and ASHP. Authorized to administer vaccines. (Age restriction: 18+ with protocol, 11-17 with prescription).	Yes
MS	Yes	Yes	Yes	Yes. Emergency order was declared on March 10, 2020. Clarified that pharmacists can work longer than 12 hours during the emergency. States that the board will not enforce pharmacist-to-technician ratios during the emergency. Authorized to administer vaccines. (Age restriction: 9+).	No
MI	Yes	No	Yes	Yes. Emergency orders was declared on March 10, 2020. Authorized licensed pharmacists to provide Medical Services for routine health maintenance, chronic disease sates, or similar conditions without physician supervision. Authorized to administer vaccines.	Yes
MN	Yes	No	Yes	Yes. Emergency orders was declared on March 13, 2020. Authorized to administer vaccines. (Age restriction: 6+).	Yes
MS	Yes	Yes	Yes. Board approves agreement and CPA may occur between multiple pharmacists and multiple physicians.	Yes. Emergency order was declared on March 14, 2020. Pharmacist may dispense one-time emergency refill of NON-CONTROLLED medication during any State of Emergency declared by the Governor. Authorized to administer vaccines.	Yes
MO	Yes	Yes	Yes	Yes. Authorized to administer vaccines.	Yes
MT	Yes	Yes	Yes. State specific requirements.	Yes. Emergency order was declared on March 12, 2020. The Board of Pharmacy has issued a temporary emergency rule, MAR 24-174-75, to suspend the requirements for monthly in-person inspections of Telepharmacy sites until August 5, 2020, in response to the COVID-19 pandemic. Authorized to administer vaccines. (Age restriction: 7+).	Yes
NE	Yes	No	Yes	Yes. Allowed pharmacists and pharmacy technicians in good standing and free from disciplinary action in other states where they are licensed to practice in Nebraska. Authorized to administer vaccines.	Yes
NV	Yes	No	Yes	Yes. Emergency order was declared on March 12, 2020. Remote order entry temporarily authorized for all pharmacy personnel, pursuant to board guidance. Authorized to administer vaccines.	Yes
**State**	**Qualified Pharmacist permitted to Initiate, Modify, and/or Discontinue Therapy**	**Additional Requirements**	**Implementation (Updated 01/05/2021)**	**COVID-19 Action (Updated on 01/05/2021)**	**Pharmacist Provider Status (Updated 1/5/2021)**

NH	Yes	Yes	Yes. Depending upon the complexity of the services being provided, the pharmacist may be required to have additional credentials or training and shall demonstrate the receipt of approval by the board of pharmacy.	Yes. Emergency order was declared on March 13, 2020. Authorized pharmacists and pharmacies to compound and sell hand sanitizer over the counter. Authorized to administer vaccines. (Age restriction: 7+).	Yes
NJ	Yes	Yes	Yes, with additional requirements.	Yes. Emergency order was declared on March 09, 2020. Authorized to administer vaccines. (Age restriction: 18+).	Yes
NM	Yes	Yes	Yes. A pharmacist may submit the application with the initial application for certification or as a separate application after becoming certified and registered as a pharmacist clinician. Only a registered pharmacist clinician with current protocols, registered with the New Mexico medical board or the New Mexico board of osteopathic medical examiners, may exercise prescriptive authority.	Yes. Authorized emergency prescription adaptation with certain stipulations including dose, dosage form, and directions for use. Did not include therapeutic substitution. Authorized to administer vaccines.	Yes
NY	Yes	Yes	Yes	Yes. Emergency order was declared on March 07, 2020. Authorized unlicensed individuals to collect specimens for COVID-19 testing, after completing training deemed adequate by the Commissioner of Health. Authorized to administer vaccines. (Age restriction: 18+).	No
NC	Yes	Yes	Yes	Yes. Allowed out-of-state licensed healthcare providers to practice in North Carolina. Out-of-state pharmacists and technicians must obtain an NABP passport to practice in North Carolina during the declared state of emergency. Authorized to administer vaccines.	Yes
ND	Yes	Yes	Yes. Collaborative agreements are regulated under "Limited Prescriptive Practices." CPA may be between multiple physicians and multiple pharmacists.	Yes. Emergency order was declared on March 13, 2020. Authorized to administer vaccines.	Yes
**State**	**Qualified Pharmacist permitted to Initiate, Modify, and/or Discontinue Therapy**	**Additional Requirements**	**Implementation (Updated 01/05/2021)**	**COVID-19 Action (Updated on 01/05/2021)**	**Pharmacist Provider Status (Updated 1/5/2021)**

OH	Yes	Yes	Yes	Yes. Emergency order was declared on March 09, 2020. Authorized remote order entry, the compounding and sale of hand sanitizer, and reuse of PPE for sterile compounding. Authorized to administer vaccines. (Age restriction: 13+).	Yes
OK	Yes	No	Yes	Yes. Declared state of emergency. Remote pharmacy practice for pharmacists and technicians was only permitted upon submission of a waiver on a case-by-case basis.	No
OR	Yes	No	Yes	Yes. Emergency order was declared on March 08, 2020. For the declared emergency time frame only, pharmacies may use remote processing functions where pharmacy personnel can perform limited functions from secure off-site, non-pharmacy location where a pharmacist may provide "remote monitoring" for prescription order entry, other data entry and insurance processing of prescriptions and medication orders. Authorized to administer vaccines. (Age restriction applied).	Yes
PA	Yes	No	Yes	Yes. No signature required for delivery/receipt of prescriptions. Pharmacists authorized to order, administer, and communicate results of Covid-19 tests. Does NOT waive or exempt CLIA regulations. Authorized to administer vaccines. (Age restriction: 18+).	No
RI	Yes	Yes	Yes, with additional training.	Yes. Emergency order was declared on March 13, 2020. Temporarily suspended the requirement to have in-person contact for patient counseling in order to allow patient counseling to be performed by telephonic consultation. Authorized to administer vaccines. (Age restriction: 18+).	Yes
SC	Yes	No	Yes	Yes. Emergency order was declared on March 13, 2020. Suspended enforcement of the prohibition on remote order entry with certain conditions. Authorized to administer vaccines. (Age restriction: 18+).	Yes
SD	Yes	No	Yes	Yes. Emergency order was declared on March 13, 2020. Authorized out-of-state licensed pharmacists to practice in South Dakota with an NABP Passport. Authorized to administer vaccines.	No
TN	Yes	No	Yes	Yes. Allowed pharmacy technicians and pharmacists to complete computer-based processing of prescriptions at alternative locations. Authorized to administer vaccines.	Yes
TX	Yes	Yes	Yes	Yes. Emergency order was declared on March 13, 2020. Temporarily suspended the requirement to have in-person contact for patient counseling in order to allow patient counseling to be performed by telephonic consultation. Authorized to administer vaccines. (Age restriction: Procedural requirement if under 14).	Yes
UT	Yes	No	Yes	Yes. Allowed the compounding and sale of certain alcohol-based hand sanitizer products by Utah-licensed pharmacies. Authorized to administer vaccines.	Yes
**State**	**Qualified Pharmacist permitted to Initiate, Modify, and/or Discontinue Therapy**	**Additional Requirements**	**Implementation (Updated 01/05/2021)**	**COVID-19 Action (Updated on 01/05/2021)**	**Pharmacist Provider Status (Updated 1/5/2021)**
VT	Yes	No	Yes	Yes. Emergency orders was declared on March 13, 2020. Authorized to administer vaccines. (Age restriction: 18+).	Yes

VA	Yes	No	Yes	Yes. Authorized pharmacists or pharmacy technicians currently licensed in another state to practice in an affected area of the Commonwealth or to provide central/remote order processing or order verification services on behalf of an out-of-state pharmacy. Authorized to administer vaccines. (Age restriction: 18+ and with prescription for minors).	Yes
WA	Yes	No	Yes. A Collaborative Drug Therapy Agreement is permitted.	Yes. Emergency order was proclaimed on February 29, 2020. Authorized to administer vaccines.	Yes
WV	Yes	Yes	Yes, with additional requirements	Yes. Permitted licensed pharmacy professionals (pharmacists, pharmacy interns, and pharmacy technicians/pharmacy technician trainees) to process prescriptions and medication orders from remote locations. Authorized to administer vaccines.	Yes
WI	Yes	No	Yes	Yes. Authorized pharmacists who are licensed in good standing in another state to practice in Wisconsin, provided they apply for a temporary license within 10 days of beginning work in Wisconsin. Authorized to administer vaccines. (Age restriction: 6+).	No
WY	Yes	No	Yes. Any licensed pharmacist, in any practice setting, can participate in a CPA. No extra training required.	Yes. Emergency order was declared on March 13, 2020. Authorized to administer vaccines.	Yes
